# Decolonise oral health care: calling for a rights-based, accountability framework approach

**DOI:** 10.3389/froh.2025.1539846

**Published:** 2025-02-28

**Authors:** Moréniké Oluwátóyìn Foláyan, Madison Cachagee, Brianna Poirier, Joelle Booth, Patricia Neville, Arish Naresh, Eleanor Fleming

**Affiliations:** ^1^ Department of Child Dental Health, Obafemi Awolowo University, Ile-Ife, Nigeria; ^2^ The Africa Oral Health Network (AFRONE), Alexandria University, Alexandria, Egypt; ^3^ Oral Health Initiative, Nigerian Institute of Medical Research, Lagos, Nigeria; ^4^ Indigenous Oral Health Unit, Adelaide Dental School, University of Adelaide, Adelaide, SA, Australia; ^5^ Peninsula Dental School, Faculty of Health, University of Plymouth, Plymouth, United Kingdom; ^6^ Bristol Dental School, University of Bristol, Bristol, United Kingdom; ^7^ College of Health and Medicine, University of Tasmania, Hobart, TAS, Australia; ^8^ Health New Zealand Tairawhiti, Gisborne, New Zealand; ^9^ University of Maryland School of Dentistry, Baltimore, MD, United States

**Keywords:** indigenous health, health inequities, colonial healthcare models, Western medical paradigms, marginalised communities, cultural competency, rights-based approach, indigenous knowledge systems

## Abstract

This paper explores how colonisation has shaped oral healthcare and oral health inequities across Indigenous populations globally. It highlights how colonial healthcare models, which prioritise Western medical paradigms, often marginalise Indigenous knowledge and practices. For Indigenous communities, such as Aboriginal Australians, Māori, and those in the Global South, oral health disparities stem from historical and ongoing structural violence, socioeconomic barriers, and limited access to culturally appropriate care. The authors argue for a decolonisation framework in global oral health that shifts power, accountability, and respect toward Indigenous and marginalised communities. A rights-based, accountability-informed decolonisation framework seeks to address historical and ongoing oral health inequities, integrating a view that oral health is a human right and demands that governments and health systems rectify the disparities. It emphasises culturally relevant care and inclusive policymaking, fostering solidarity and systemic change to create equitable and effective oral healthcare for all populations. We propose that deliberate actions need to be taken to centre power redistribution, accountability, and respect in global oral health, moving away from Euro-American-centric frameworks to create an equitable, culturally responsive oral healthcare system. Our calls to action include the need for self-reflection within the field to dismantle entrenched colonial ideologies and prioritise Indigenous leadership and knowledge. Effective allyship should involve collaboration driven by the needs of communities, with institutions accountable for reducing exclusionary practices. By “learning to unlearn” traditional frameworks, the oral health community can build a system that genuinely addresses health disparities and supports justice and equity worldwide.

## Introduction

Colonialism profoundly impacted the development of healthcare systems worldwide. By design, colonial societies prioritise the needs of settlers through the oppression of colonised peoples. Colonial healthcare systems were founded upon the belief that colonised peoples were inferior to settlers, and these healthcare systems remain structured by settler colonial ideologies (1–3). The continued structuring of healthcare models using Western paradigms further marginalises Indigenous Peoples through multiple mechanisms, such as the subalternation of Indigenous knowledge systems and healing practices that remain integral to Indigenous communities. This has implications for growing inequalities in the global disease burden.

In the Global North, Indigenous populations such as Aboriginal and Torres Strait Islander communities in Australia, Māori communities in Aotearoa/New Zealand, and Indigenous communities on Turtle Island, North and Central America (First Nation, Métis, Inuit, Alaskan Native, and American Indian) experience a higher burden of oral disease than non-Indigenous populations (4–9). The high level of untreated dental caries, lower number of restored teeth and high prevalence of periodontal disease (10) are reflective of systemic inequities. Factors such as limited access to healthcare services (11), economic disparities (12), cultural disconnection (13), stigmatisation (14), and historical trauma (15) contribute to the prevalence of oral health issues. In addition, the lack of culturally appropriate dental care and preventive measures exacerbates the situation (16). This disparity is directly related to the impact of colonialism and its intersection with racism and intergenerational trauma (17).

In the Global South, the oral disease burden is skewed to countries with low economic resources; and, within the country, to those with low socioeconomic status. This is reflected in the oral health data of the 47 countries in the World Health Organization (WHO) Africa Region, of which 43 low- to low-middle-income countries has increased oral disease burden over the last 30 years. Currently 44% of the population in the African WHO region suffers from oral diseases (18). The oral health disparities in the Global South are deeply rooted in the legacy of colonialism, which disregarded traditional oral health practices in favour of Western methods (3). In addition, the influx of migrant workers supporting colonial industries brought about changes in dietary patterns that contributed to an increase in dental caries (19). Today, the 46 of the 47 countries in the World Health Organization Africa Region that had experienced colonisation, continue to grapple with the political, social, and economic consequences of these historical disruptions, facing underfunded healthcare systems (20), inaccessible oral health services (21), and persisting Western healthcare models that frequently do not align with local needs.

Similarly, Pacific Island Countries and Territories (PICTs) experience very high levels of dental caries and periodontal disease, increasing over the years. Over the past 40 years, the inequities in population oral health have widened, despite technologies and innovations that have narrowed some disparities in other parts of the world. Western diets and movement away from traditional ways of living have played a role in this change (22). In the Pacific region, the transition of the Indigenous Peoples in Fiji to a dependency on facility-based oral health care over traditional oral health self-management practices exemplifies the effect of colonial ideologies on oral health practices and self-determination (22).

The shift to facility-based oral health care undermined the use of local plants, natural remedies, and community-based health approaches. This led to losing valuable knowledge, cultural heritage, and self-determination in oral health as indigenous and local knowledge are poorly integrated. It fostered dependency on biomedical models of health that focus on treating symptoms rather than the Indigenous holistic approach encompassing physical, mental, and spiritual well-being, and ultimately failing to address the root causes of health issues (23–26). Barriers to health and oral healthcare access were further created due to geographic isolation, financial constraints, and inadequate transportation (22, 26–28). Facility-based care also often lacks cultural sensitivity, making it difficult for Indigenous Peoples to feel understood or respected, which discourages them from seeking care. In addition, the dependency on the facility for care reinforces power imbalances, as healthcare decisions are frequently made by providers who may not fully understand or respect Indigenous cultural contexts.

The imposed Euro-American-centric approach to healthcare delivery on Indigenous populations and other marginalised groups create healthcare landscapes that are misaligned with the cultural and healing needs of the populations. The poor recognition of the local life philosophy and context contributed to the enforcement of a Euro-American-centric approach to oral health care for Indigenous Peoples (29). Settler colonialism sustains harmful stereotypes of Indigenous Peoples as disempowered communities, with narratives that reinforce dependence and vulnerability that are validated and perpetuated through policies (4). By framing Indigenous Peoples as mere recipients of care, rather than active participants in it, settler colonialism attempts to restrict Indigenous Peoples' assertion of self-determination as primary agents in their care (30).

Moreover, in the removal of self-determination, colonisation also positioned Indigenous Peoples to be the subjects of tests to advance care and research, often without their consent (31). The contraception study trials in Puerto Rico serve as an example of settler colonialism's impact on the healthcare landscape and science to advance care innovation (32, 33). Colonisation thus drives health inequities, not only due to the loss of cultural identity but also due to the active oppression of Indigenous populations by colonisers for their own gain and power maintenance (34).

The decolonisation framework is required to address entrenched health inequities—including oral health inequities—that are perpetuated by settler colonialism. For example, during the 2024 mpox outbreak, decolonisation provided a framework for the Africa Centers for Disease Control and Prevention to take leadership for the public health response in Africa by declaring the first-ever Public Health Emergency of Continental Security due to the rising mpox cases, mortality spreading to non-endemic countries (35). The call was also for the restructuring of global health supply chains to prioritise Africa's vaccine access (36, 37) and fostered a partnership model where African governments shaped policy decisions, resource allocations, and responses (38). This shift came after the neglect Africa faced during the 2022 mpox outbreak, when vaccines were mainly accessible to high-income countries, despite Africa being the epicenter (39). The July 2022 mpox Public Health Emergency of International Concern was removed in May 2023 while cases were still rising in the Democratic Republic of Congo, the epicentre of the infection (40).

The mpox outbreak, similar to oral health, illustrates how a decolonisation lens is needed to support effective public oral health responses and to prioritise equitable access to healthcare resources, particularly for historically marginalised populations. This approach challenges existing global health frameworks that often favour high-income countries and underscores the importance of local leadership, community-centered strategies, and resource sovereignty to address oral health inequities at their roots. By applying a decolonisation framework, public oral health can shift towards self-determined, culturally relevant interventions that more effectively meet the unique needs of African communities.

Applying the decolonisation framework to the global oral health response is important, knowing that the burden of oral diseases affecting over 3.5 billion people globally is skewed to low- and middle-income countries (LMICs) (41). In addition, the global caseload of oral disease surpasses the estimated population growth rate between 1990 and 2019, indicating that the current measures and policies to prevent and control oral diseases have explicitly failed to the disadvantage of vulnerable populations (42). The dominance of global actors in decision-making for local actions, excluding local knowledge, cultural practices, and traditional oral healthcare approaches, has contributed to the observed skewness in the oral disease burden. Western-led research and policies often overlook the specific needs of LMICs, and this is further disadvantageous to these regions in addressing oral health inequities.

Oral health inequities resulting from colonisation, and sustained by settler colonialism, are a form of structural violence (43). Structural violence refers to subtle, often invisible, systematic ways in which social structures harm or disadvantage individuals and often have no one specific person who can (or will) be held responsible. It prevents individuals, groups and societies from reaching their full potential by creating barriers to meeting basic needs or achieving one's potential (44). Colonisation prevents populations from equitable access to dental care and treatment, leading to higher rates of untreated dental caries, periodontal disease, and other oral health issues. It results from the policy and governance that perpetuate colonial ideologies that fail to recognise or support Indigenous health practices, resulting in a lack of investment in community-led health initiatives. This lack of representation and advocacy reinforces structural violence by maintaining oral health disparities.

Viewing oral health inequities through the lens of structural violence emphasises the need for systemic change. There is a moral and professional obligation for the global dental professionals and the oral health research community to recognise oral health inequities driven by colonialism, as an urgent call for action (45, 46). By disassembling settler supremacy and dismantling colonial systems that directly create and sustain oral health inequities, decolonial frameworks and approaches can develop transformative and inclusive approaches that disrupt oral disease patterns (47).

### Power, accountability, and decolonisation global oral health

Established and championed by Indigenous and marginalised leaders in their pursuits of health justice, decolonisation is a disruptive rather than complementary approach to human rights and self-determination (48, 49). Decolonisation de-centres colonial ideologies that continue to oppress Indigenous and marginalised communities and requires us to challenge the entrenched inequities within oral health globally (49). A global decolonial framework for oral health must recognise and value Indigenous knowledge and leadership; address the structural inequities that contribute to oral health disparities; prioritise local policy reform that promotes equitable resource distribution; and establish accountability mechanisms that hold healthcare systems, researchers, and policymakers responsible for addressing oral health disparities. It must be recognised that research and biomedical care are part of colonial processes and, therefore, require targeted efforts and structural change that prioritise decolonial values and necessitate decolonial practices (47).

At the heart of decolonisation is power. The glaring power differentials, for example, the Global North and Global South, often results in solutions to address oral health inequities with the unintended consequence of replicating colonial relationships rather than supporting transformative work to address inequities. Despite the long-standing evidence of oral health inequities in the Global North among ethnic minorities and populations with low socioeconomic status (50, 51), the World Health Organization failed to include oral health in its agenda until 2021 (52)—which limited global attention and action on oral health inequities in low- and middle-income countries.

Decolonisation requires that power be niched with accountability. Accountability requires power redistribution to generate relevant knowledge to develop meaningful policies and programmes to reduce health disparities (53). It requires healthcare providers, researchers, and policymakers to be answerable to the historical and persisting disparities in oral health. It is, however, not just about recognising the damage caused by the dominance of Western-centric practices; it's about taking proactive steps to correct these inequities. It requires intentional efforts to free and make local and Indigenous knowledge, practices, and culture visible from the prevailing power structures (54) by considering diverse perspectives, particularly those of individuals whose existence is marginalised and deemed insignificant, in decision-making (55). This can ensure the development of inclusive policies, the fair allocation of resources, and the continuous involvement of communities in the decision-making process.

With this accountability-informed decolonisation framework, a rights-based perspective can be used to build a monitoring schema for global actions. A rights-based perspective posits that failing to achieve progress in realising the highest attainable standard of oral health for a population is a health system failure and a human rights violation (56–58). Integrating a rights-based perspective within an accountability-informed decolonisation framework is a powerful approach to addressing health disparities and promoting social justice. It offers a transformative approach to oral health equity by holding systems accountable for upholding the right to oral health and centering the experiences of marginalised communities. This makes building a more just and equitable oral health landscape possible by seeking to rectify historical injustices and promote a future where all individuals can realise their right to health.

A rights-based perspective recognises oral health as a fundamental human right, as outlined in the Universal Declaration of Human Rights and the International Covenant on Economic, Social and Cultural Rights. It asserts that everyone has the right to achieve the highest standard of oral health, which encompasses timely access to healthcare and essential determinants like clean water, nutritious food, and adequate housing. When populations cannot attain this standard, it reflects a violation of human rights and exposes systemic inequities affecting vulnerable groups. This perspective shifts responsibility from individual blame to systemic accountability, which holds the governments and health systems accountable for equitable oral health resource access and to redress structural violence. By integrating decolonisation into this framework, redress mechanisms must address the ongoing impacts of colonialism on oral health practices and empower communities to reclaim their health sovereignty, ultimately improving oral health outcomes and equity in care access.

At the global level, a rights-based, accountability-informed decolonisation framework enables the creation of a comprehensive monitoring schema for assessing global oral health actions. This schema would include metrics to evaluate oral health outcome disparities among marginalised populations, involve communities in developing culturally relevant monitoring tools, and establish accountability structures for governments and organisations that fail to meet their oral health-related obligations. This may involve reporting requirements, feedback mechanisms, and avenues for redress. By empowering marginalised voices, the framework challenges the power dynamics that often silence these populations in health systems. It emphasises that oral health is a collective right, fostering solidarity among communities, advocates, and policymakers to mobilise resources for health equity. In addition, it necessitates an intersectional analysis that recognises how discrimination based on race, gender, class, and disability intersects to influence oral health outcomes, ensuring that the monitoring schema addresses these complexities in health disparities.

This way, accountability can reinforce equally shared respect between Global North and Global South collaborators. Respect means moving away from the idea that Western methods are the gold standard and embracing a more pluralistic approach that includes traditional practices and cultural safety (59). This shift requires a genuine commitment to understanding and integrating oral health practices that have been passed down through generations within Indigenous and marginalised communities. By valuing these practices, the oral healthcare system can provide more culturally appropriate care, essential for building trust and ensuring better oral health outcomes.

### What next for decolonizing global oral health

Decolonising oral health care requires redistributing power and creating accountability frameworks at the global and local levels to promote a more equitable and effective care system. This may require the inclusion of non-dental practitioners, such as Indigenous health workers, in oral health care promotion and provision to create a culturally safe knowledge-sharing environment that empowers self-determination of oral health (60). This approach can facilitate recognising oral health as a fundamental component of overall health, moving beyond the restrictive norms and philosophical debates that have historically marginalised oral health in global health discussions (61). By doing so, it positions the right to oral health within a broader framework of health rights, addressing the systemic barriers that have long excluded oral health from being considered an integral part of general health and human rights considerations. This requires changes in policy and practice, a commitment to truth and reconciliation, and addressing the systemic inequities that have led to disparities in oral health outcomes.

At the country level, integrating Indigenous oral health practices with Western medical care requires a respectful, balanced approach valuing both systems. This integration enhances patient outcomes by enabling Indigenous communities to access culturally familiar care alongside modern dental and healthcare advancements. Developing a referral network between Indigenous practitioners and Western providers would foster trust, allowing patients to transition smoothly between systems based on needs and preferences. Successful integration requires governmental support, including frameworks recognising and funding Indigenous practitioners. Cross-training programmes for Indigenous and Western providers would deepen mutual understanding, while community involvement in design and implementation ensures relevance and acceptance. Structural barriers, such as insufficient funding or limited legal recognition, must be addressed to achieve equity. This approach promises a more inclusive oral healthcare system that respects cultural diversity and, if well-funded and community-driven, could significantly improve accessibility and quality of oral healthcare for Indigenous populations. Effective integration requires robust monitoring to assess patient satisfaction, health improvements, and referral success rates, with community feedback continuously refining the process for sustained impact.

As research directly informs care provision and resource allocation, decolonising research methodologies is essential to a decolonial oral health agenda. By design, decolonial research pursuits create sustainable change because they are community-led and align with community ways of knowing, being, and doing. Decentering researcher aims and dismantling colonial agendas upheld by academic institutions is required of any decolonial project. The use of participatory research methods is one potential pathway to decolonise research because of the opportunity for research participants to actively disrupt the power symmetries that traditionally define knowledge production (62). For example, the ring vaccination strategy used in Sierra Leone for the testing of the Ebola vaccine was a departure from the randomized control trial upheld as the golden standard of practice. The ring vaccination methodology aligns with community-based health norms, prioritising collective well-being over the individualistic approach of clinical trials, and yet maintained standards required for the conduct of clinical trials (63). The ring vaccination strategy was appropriate for communitarian societies that would find it difficult to justify why some individuals would receive experimental therapies that had good chances of reducing the risk of death while others, equally at risk, would not have the same opportunity in a system where collective decisions are made based on shared community values and the well-being of the group rather than individual risk-benefit calculations. This approach ensured ethical acceptability while still adhering to rigorous scientific standards for evaluating vaccine efficacy and safety. Randomised clinical trials with placebo controls could create ethical dilemmas that conflict with the community's social values (64).

## Conclusion: a call to action

Power redistribution, accountability, respect and disruption of colonial ideologies must be at the center of the global oral health planning, implementation, monitoring and evaluation processes, guiding our efforts to create a more equitable and culturally responsive oral health care system. By challenging the entrenched Euro-American-centric frameworks that have dominated oral health research and practice, our openness to “learning to unlearn” (65) the conventional canon will provide us with the means and opportunity to “relearn” (65) as an oral healthcare profession. It would also enable the professionals in the oral health field and its allies to address the disparities that continue to affect Indigenous and marginalised populations around the world.

It is a call for a self-reflective process—to examine our positions, privileges, and connections to colonial systems that continue to shape oral health practices and knowledge—while recognising the dynamics of coloniality in the production of dominant thoughts (66, 67), and the advantages derive from the entrenched structures and systems. Actors and players must be willing to confront how the education and institutions we trust are often embedded within a colonial legacy, and acknowledge how these systems maintain barriers to equity. This self-awareness is the first step for meaningful allyship, emphasising supportive roles as enablers rather than leaders in spaces where privileges had dominated.

As Abimbola and Pai urge (68), we also need to advocate for change within academic and health structures that currently reinforce a Global North dominance, often at the expense of relationships with the communities served. Allyship means fostering genuine collaboration, where the agendas are driven by Indigenous communities' needs rather than by other actors' priorities. These actions should extend to holding institutions accountable for dismantling exclusionary practices that deny representation to marginalised groups. Embracing an approach to research with and clinical care for Indigenous communities that centers on their leadership and priorities—rather than those of outside agendas—marks a crucial shift towards empowerment, accountability, and respect.

Regional calls for action, such as the Suva declaration and the WHO global action plan for Fuji, can be powerful tools for change (69, 70) as they can provide a renewed focus on oral health improvement in regions. These calls can, however, be strengthened by being bold and explicitly stating that Indigenous oral health practices should be strongly recognised in mainstream oral health prevention and promotion.

Furthermore, there is the need to critically assess the positioning of global health and oral health organisations, many Euro-American-centric and white-dominated, and push for a more equitable redistribution of power and resources. Decolonising global health requires dismantling all forms of supremacy through prioritising the redistribution of power and enforcing the disruption of colonial ideologies through frameworks that promote accountability and respect. Embracing the process of “learning to unlearn” conventional frameworks in global oral health will allow the collective players in the field to “relearn” through diverse perspectives. Such openness offers the oral health field—and all its allies—the opportunity to address disparities that continue to harm Indigenous and marginalised communities worldwide, thereby building a more equitable and culturally responsive oral health care system. This is a matter of justice and a necessary step towards creating a healthier and more equitable world.

## Data Availability

The original contributions presented in the study are included in the article/Supplementary Material, further inquiries can be directed to the corresponding author.

## References

[1] Tilley H. Medicine, empires, and ethics in colonial Africa. AMA J Ethics. (2016) 18(7):743–53. 10.1001/journalofethics.2016.18.7.mhst1-1607 27437825

[2] Wispelwey B Tanous O Asi Y Hammoudeh W Mills D. Because its power remains naturalised: introducing the settler colonial determinants of health. Front Public Health. (2023) 11:1137428. 10.3389/fpubh.2023.1137428 37533522 PMC10393129

[3] Abdullahi AA. Trends and challenges of traditional medicine in Africa. Afr J Tradit Complement Altern Med. (2011) 8(5 Suppl):115–23. 10.4314/ajtcam.v8i5S.5 22754064 PMC3252714

[4] Schuch HS Haag DG Kapellas K Arantes R Peres MA Thomson WM The magnitude of indigenous and non-indigenous oral health inequalities in Brazil, New Zealand and Australia. Commun Dent Oral Epidemiol. (2017) 45(5):434–41. 10.1111/cdoe.12307 28509420

[5] Nath S Poirier BF Ju X Kapellas K Haag DG Ribeiro Santiago PH Dental health inequalities among indigenous populations: a systematic review and meta-analysis. Caries Res. (2021) 55(4):268–87. 10.1159/000516137 34107490 PMC8491513

[6] Bramley D Hebert P Tuzzio L Chassin M. Disparities in indigenous health: a cross-country comparison between New Zealand and the United States. Am J Public Health. (2005) 95(5):844–50. 10.2105/AJPH.2004.040907 15855464 PMC1449267

[7] Mangla A Agarwal N. Clinical practice issues in American Indians and Alaska natives. 2023 may 29. In: StatPearls. Treasure Island (FL): StatPearls Publishing (2024). 34033363

[8] Tynan A Walker D Tucker T Fisher B Fisher T. Factors influencing the perceived importance of oral health within a rural aboriginal and torres strait islander community in Australia. BMC Public Health. (2020) 20(1):514. 10.1186/s12889-020-08673-x 32303214 PMC7164228

[9] Carstairs C Mosby I. Colonial extractions: oral health care & indigenous people in Canada, 1945–1979. Can Hist Rev. (2020) 101:192–216. 10.3138/chr.2018-0097

[10] Peres MA Macpherson LMD Weyant RJ Daly B Venturelli R Mathur MR Oral diseases: a global public health challenge. Lancet (London, England). (2019) 394(10194):249–60. 10.1016/S0140-6736(19)31146-8 31327369

[11] Davy C Harfield S McArthur A Munn Z Brown A. Access to primary health care services for indigenous peoples: a framework synthesis. Int J Equity Health. (2016) 15:163. 10.1186/s12939-016-0450-5 27716235 PMC5045584

[12] Organisation for Economic Co-operation and Development. Indigenous communities. Available online at: https://www.oecd.org/en/topics/sub-issues/indigenous-communities.html#:∼:text=Although%20Indigenous%20peoples%20make%20up,in%20rural%20and%20remote%20areas (Accessed: October 15, 2024).

[13] Shepherd SM Delgado RH Sherwood J Paradies Y. The impact of indigenous cultural identity and cultural engagement on violent offending. BMC Public Health. (2018) 18(1):50. 10.1186/s12889-017-4603-2. Erratum in: *BMC Public Health*. (2017) 17(1):736. PMC5525355 28738789

[14] Poirier B Hedges J Smithers L Moskos M Jamieson L. “I feel like the worst mother in the world”: neoliberal subjectivity in indigenous Australian oral health. SSM-Qual Res Health. (2022) 2:100046. 10.1016/j.ssmqr.2022.100046

[15] Smallwood R Woods C Power T Usher K. Understanding the impact of historical trauma due to colonisation on the health and well-being of indigenous young peoples: a systematic scoping review. J Transcult Nurs. (2021) 32(1):59–68. 10.1177/1043659620935955 32567510

[16] Tynan A Walker D Tucker T Fisher B Fisher T. Managing oral health care and prevention: the experience of aboriginal and torres strait islanders living in a rural community in Queensland, Australia. Aust J Rural Health. (2022) 30(2):228–37. 10.1111/ajr.12853 35196414 PMC9306970

[17] Jamieson L Hedges J McKinstry S Koopu P Venner K. How neoliberalism shapes indigenous oral health inequalities globally: examples from five countries. Int J Environ Res Public Health. (2020) 17(23):8908. 10.3390/ijerph17238908 33266134 PMC7730877

[18] World Health Organization. Africa burdened with largest global increase of oral diseases. (2023). Available online at: https://www.afro.who.int/news/africa-burdened-largest-global-increase-oral-diseases (Accessed: October 30, 2024).

[19] van der Merwe AE Steyn M Maat GJR. Dental health of 19th century migrant mineworkers from kimberley, South Africa. Int. J. Osteoarchaeol. (2011) 21:379–90. 10.1002/oa.1143

[20] Josefczyk M. The State of Oral Health on the African Continent. A Senior Thesis submitted in partial fulfillment of the requirements for graduation in the Honors Program Liberty University. (2015). Available online at: https://digitalcommons.liberty.edu/cgi/viewcontent.cgi?referer=&httpsredir=1&article=1578&context=honors (Accessed: October 30, 2024).

[21] Sindhu R Manipal S Mohan R Bharathwaj VV Lalitha ND Prabu D. Perceived oral health beliefs, traditional practices, and oral health status of nomads of tamilnadu: a cross-sectional study. J Family Med Prim Care. (2020) 9(1):131–5. 10.4103/jfmpc.jfmpc_618_19 32110578 PMC7014911

[22] Doherty MAH Blinkhorn AS Vane ES. Oral health in the pacific islands. Int Dent J. (2010) 60(2):122–8. 10.1922/IDJ_2242Doherty07 20476718

[23] Tukuitonga C Shiu RN Akauola S Chutaro E Fried C. Sustainability and Resilience in Pacific Island Health Systems, Center for Asia-Pacific Resilience and Innovation (CAPRI). Available online at: https://www3.weforum.org/docs/WEF_PHSSR_CAPRI_Pacific_Islands_2024.pdf (Accessed: October 27, 2024).

[24] Tatui L McCool J Nosa V. Rethinking and establishing a dental collaboration in the pacific region. Pac Health Dialog. (2018) 21(2):108–10. 10.26635/phd.2018.921

[25] Arish N. Oral health inequalities and the important role of mid-level dental providers. Malaysian Dental Therapy Conference, Kuala Lumpur (2019).

[26] Venina W. Fiji Public health experiences, services and challenges. Fiji Oral Health Conference Proceedings. Naji. Fuji: Fuji Oral Health Association (2024) p. 10.

[27] Maimanuku L Piukala S Tatui L Tiim K Benzian H. Oral diseases and NCDs in the western pacific region need to be prioritised. Lancet Reg Health West Pac. (2024) 47:101099. 10.1016/j.lanwpc.2024.101099 38827184 PMC11140791

[28] Maimanuku L Piukala S Tatui L Tiim K Benzian H. New leadership for WHO western pacific region: a call to prioritise oral health in the pacific islands. Front Public Health. (2024) 12:1388117. 10.3389/fpubh.2024.1388117 39040860 PMC11260744

[29] Boodman E. Medical colonialism and the power to care: unsettling participatory inclusion in the settler-state care paradigm. Hypatia. (2023) 38(2):330–52. 10.1017/hyp.2023.24

[30] Macdonald NE Stanwick R Lynk A. Canada's shameful history of nutrition research on residential school children: the need for strong medical ethics in aboriginal health research. Paediatr Child Health. (2014) 19(2):64. 10.1093/pch/19.2.64 24596474 PMC3941673

[31] Marks LV. Sexual Chemistry: A History of the Contraceptive Pill. New Haven: Yale University Press (2001).

[32] Tone A. Devices and Desires — a History of Contraceptives in America. New York: Hill and Wang (2001).

[33] Ramírez de Arellano AB Seipp C. Colonialism, Catholicism, and Contraception: A History of Birth Control in Puerto Rico. Chapel Hill: University of North Carolina Press (1983).

[34] Abimbola S Asthana S Montenegro C Guinto RR Jumbam DT Louskieter L Addressing power asymmetries in global health: imperatives in the wake of the COVID-19 pandemic. PLoS Med. (2021) 18(4):e1003604. 10.1371/journal.pmed.1003604 Erratum in: PLoS Med. 2021 Jun 7;18(6):e1003667. 33886540 PMC8101997

[35] Africa CDC. Africa CDC declares mpox a Public Health Emergency of Continental Security, mobilising resources across the continent. Published online August 13, 2024. Available online at: https://africacdc.org/news-item/africa-cdc-declares-mpox-a-public-health-emergency-of-continental-security-mobilizing-resources-across-the-continent/ (Accessed: October 25, 2024).

[36] Africa CDC. Declaration of Public Health Emergency of Continental Security. Available online at: https://africacdc.org/wp-content/uploads/2024/08/Explainer-Declaration-of-Public-Health-Emergency-of-Continental-Security-EN.pdf (Accessed: October 25, 2024).

[37] Boum Y 2nd Ndembi N. Mpox needs a locally tailored global response. Br Med J. (2024) 386:q2094. 10.1136/bmj.q2094 39332834

[38] Africa CDC. Mpox Response Plan Envisions Support Across 22 Countries. Available online at: https://africacdc.org/news-item/mpox-response-plan-envisions-support-across-22-countries/#:∼:text=The%20finalised%20continental%20response%20plan,efforts%20and%20effective%20resource%20allocation (Accessed: October 25, 2024).

[39] Zumla A Rosenthal PJ Sam-Agudu NA Ogoina D Mbala-Kingebeni P Ntoumi F The 2024 public health emergency of international concern: a global failure to control mpox. Am J Trop Med Hyg. (2024) 112(1):17–20. 10.4269/ajtmh.24-0606 39406210 PMC11720770

[40] Sam-Agudu NA. Mpox Declares to Africa that Global Health is in Fact, Not global. August 16, 2024. PLOS Global Public Health. Available online at: https://speakingofmedicine.plos.org/2024/08/16/mpox-declares-to-africa-that-global-health-is-in-fact-not-global/ (Accessed: October 25, 2024).

[41] World Health Organization. Global Oral Health status Report: Towards Universal Health Coverage for Oral Health by 2030. Geneva: World Health Organization (2022). https://onlinelibrary.wiley.com/doi/full/10.1111/odi.14516

[42] Tu C Wang G Hu Z Wang S Yan Q Liu X. Burden of oral disorders, 1990–2019: estimates from the global burden of disease study 2019. Arch Med Sci. (2023) 19(4):930–40. 10.5114/aoms/165962 37560733 PMC10408023

[43] Farmer PE Nizeye B Stulac S Keshavjee S. Structural violence and clinical medicine. PLoS Med. (2006 ) 3(10):e449. 10.1371/journal.pmed.0030449 17076568 PMC1621099

[44] Galtung J. Violence, peace and peace research. J Peace Res. (1969) 6:167–91.

[45] Foláyan MO Haire B. What’s trust got to do with research: why not accountability? Front. Res Metr Anal. (2023) 8:1237742. 10.3389/frma.2023.1237742 38025960 PMC10679329

[46] Fleming E Smith CS Quiñonez CR. Centring anti-oppressive justice: re-envisioning dentistry’s social contract. Community Dent Oral Epidemiol. (2023) 51(4):609–14. 10.1111/cdoe.12854 36966445

[47] Hedges J Poirier B Soares G Haag D Sethi S Santiago PR Journeying towards decolonising aboriginal and torres strait islander oral health re-search. Community Dent Oral Epidemiol. (2023) 51:1232–40. 10.1111/cdoe.12881 37294001

[48] Tuck E Yang KW. Decolonisation is not a metaphor. Decolonization: indigeneity, education & society. Decolonization: Indigeneity, Education & Society. (2012) 1(1):1–40. 10.25058/20112742.n38.04

[49] Corntassel J. Re-envisioning resurgence: Indigenous pathways to decolonisation and sustainable self-determination. Decolonisation: Indigeneity, Education & Society. (2012). 1(1). Available online at: https://jps.library.utoronto.ca/index.php/des/article/view/18627/15550 (Accessed October 25, 2024).

[50] Nath S Sethi S Bastos JL Constante HM Kapellas K Haag D A global perspective of racial-ethnic inequities in dental caries: protocol of systematic review. Int J Environ Res Public Health. (2022) 19(3):1390. 10.3390/ijerph19031390 35162411 PMC8835154

[51] Van Nieuwenhuysen JP Carvalho JC D’Hoore W. Status of dental caries in Belgium and neighboring countries. Rev Belge Med Dent. (2002) 57(3):186–205. 12508719

[52] Lamster IB. The 2021 WHO resolution on oral health. Int Dent J. (2021) 71(4):279–80. 10.1016/j.identj.2021.06.003 34256923 PMC9275116

[53] United Nations. United Nations. Declaration on the Rights of Indigenous Peoples. General Assembly Resolution 61/295 on 13 September 2007. Available online at: https://www.culturalsurvival.org/undrip?gad_source=1&gclid=CjwKCAjwx4O4BhAnEiwA42SbVKrzYikAEo7zk_FZmVtsTDCQnNkzkBCDWgqdJ8lURdBdvHg55xLr5RoCkhEQAvD_BwE (Accessed: September 5, 2024).

[54] Emnet TW. Decolonising a higher education system which has never been colonised. Educ Philo Theory. (2021) 53(9):894–906. 10.1080/00131857.2020.1835643

[55] Ndlovu-Gatsheni SJ. Epistemic Freedom in Africa: Deprovincialization and Decolonisation. 1st ed. London: Routledge (2018). 10.4324/9780429492204

[56] Jean G Kruger E Lok V. Oral health as a human right: support for a rights-based approach to oral health system design. Int Dent J. (2021) 71(5):353–7. 10.1016/j.identj.2020.12.021 33612265 PMC9275327

[57] Secretary-General. United Nations; Geneva, Switzerland. The right of everyone to the enjoyment of the highest attainable standard of physical and mental health. 2006. Report No.: A/61/338).

[58] Tobin J. The Right to Health in International law. 1st ed. Oxford, UK: Oxford University Press (2012).

[59] Cant S. Medical pluralism, mainstream marginality or subaltern therapeutics? Globalisation and the integration of ‘Asian’ medicines and biomedicine in the UK. Soc Culture South Asia. (2020) 6(1):31–51. 10.1177/2393861719883064

[60] Poirier B Sethi S Hedges J Jamieson L. Building an understanding of indigenous health Workers’ role in oral health: a qualitative systematic review. Community Dent Oral Epidemiol. (2023) 51(2):169–79. 10.1111/cdoe.12743 35324023

[61] Paton M Naidu T Wyatt TR Oni O Lorello GR Najeeb U Dismantling the master’s house: new ways of knowing for equity and social justice in health professions education. Adv Health Sci Educ Theory Pract. (2020) 25(5):1107–26. 10.1007/s10459-020-10006-x 33136279 PMC7605342

[62] Omodan BI Dastile NP. Analysis of participatory action research as a decolonial research methodology. Soc Sci. (2023) 12(9):507. 10.3390/socsci12090507

[63] Haire BG Folayan MO. Ebola “ring” vaccine trial was ethically innovative. Am J Public Health. (2016) 106(9):e1. 10.2105/AJPH.2016.303311 27509285 PMC4981795

[64] Folayan MO Haire B. Communitarian societies and public engagement in public health. Crit Public Health. (2017) 27(1):6–13. 10.1080/09581596.2016.1252035

[65] Tlostanova MV Mignolo W. Learning to Unlearn: Decolonial Reflections from Eurasia and the Americas. Columbus, OH: Ohio State University Press (2012).

[66] Mathieu X. The dynamics of ‘civilised’ sovereignty: colonial frontiers and performative discourses of civilisation and savagery. Int Relat. (2018) 32(4):468–87. 10.1177/0047117818782612

[67] Neville P. Decolonising dental educational research: reflections from a white researcher. Adv Health Sci Educ Theory Pract. (2023) 28(5):1679–95. 10.1007/s10459-023-10228-9 37074593 PMC10113732

[68] Abimbola S Pai M. Will global health survive its decolonisation? Lancet. (2020) 396(10263):1627–8. 10.1016/S0140-6736(20)32417-X 33220735

[69] Oral Health Pacific Alliance. Suva Declaration on Improving Oral Health in the Pacific Islands Region. Fiji National University. July 20, 2023. Available online at: https://www.fnu.ac.fj/wp-content/uploads/2023/08/Suva-Declaration-2023.pdf (Accessed October 25, 2024).

[70] World Health Organization. Meeting on Health Information System Strengthening in Pacific Island Countries in Suva, Fiji. Meeting report. 27-29 June 2018.

